# Cortical Oscillations in Cervical Dystonia and Dystonic Tremor

**DOI:** 10.1093/texcom/tgaa048

**Published:** 2020-08-20

**Authors:** Christopher W Hess, Bryan Gatto, Jae Woo Chung, Rachel L M Ho, Wei-en Wang, Aparna Wagle Shukla, David E Vaillancourt

**Affiliations:** 1 Fixel Institute for Neurological Diseases, Department of Neurology, University of Florida, Gainesville, FL 32611, USA; 2 Laboratory for Rehabilitation Neuroscience, Department of Applied Physiology and Kinesiology, University of Florida, Gainesville, FL 32611, USA; 3 J. Crayton Pruitt Family Department of Biomedical Engineering, University of Florida, Gainesville, FL 32611, USA; 4 Department of Neurology, University of Minnesota, Minneapolis, MN 55455, USA

**Keywords:** dystonia, head movement, motor cortex, oscillation, sensory cortex

## Abstract

Dystonia involves sustained or repetitive muscle contractions, affects different skeletal muscles, and may be associated with tremor. Few studies have investigated if cortical pathophysiology is impaired even when dystonic muscles are not directly engaged and during the presence of dystonic tremor (DT). Here, we recorded high-density electroencephalography and time-locked behavioral data in 2 cohorts of patients and controls during the performance of head movements, upper limb movements, and grip force. Patients with cervical dystonia had reduced movement-related desynchronization in the alpha and beta bands in the bilateral sensorimotor cortex during head turning movements, produced by dystonic muscles. Reduced desynchronization in the upper beta band in the ipsilateral motor and bilateral sensorimotor cortex was found during upper limb planar movements, produced by non-dystonic muscles. In a precision grip task, patients with DT had reduced movement-related desynchronization in the alpha and beta bands in the bilateral sensorimotor cortex. We observed a general pattern of abnormal sensorimotor cortical desynchronization that was present across the head and upper limb motor tasks, in patients with and without DT when compared with controls. Our findings suggest that abnormal cortical desynchronization is a general feature of dystonia that should be a target of pharmacological and other therapeutic interventions.

## Introduction

Dystonia is a movement disorder resulting in abnormal postures and/or movements that are often accentuated by voluntary movements (Albanese et al. 2013). Its pathophysiology is poorly understood and likely heterogeneous, and abnormalities in cortical inhibition, sensorimotor integration, and plasticity have been proposed (Jinnah and Hess 2018). One hallmark of dystonia is heterogeneity in presentation of signs. Some individuals present with task-specific dystonia affecting only a few muscles, whereas other individuals present with more generalized signs affecting many axial and appendicular muscles. In addition, some forms of dystonia express tremor, whereas other forms of dystonia do not express tremor (Bhatia et al. 2018; DeSimone et al. 2019). A wealth of noninvasive neuroimaging studies have recently demonstrated widespread neuronal network-level dysfunction (Battistella et al. 2017; Burciu et al. 2017; Filip et al. 2017; Corp et al. 2019) in patients with dystonia, with the majority of this work performed using neuroimaging and functional magnetic resonance imaging (fMRI). Despite excellent spatial resolution, fMRI does not have sufficient temporal resolution to study movement-related cortical dynamics. Examining the sensorimotor frequency-specific oscillations that are time locked to the stages of movement are fundamental, because they are thought to reflect synchronized activity of neuronal populations (Pfurtscheller and Lopes da Silva 1999).

Abnormalities of movement-related cortical oscillations have been demonstrated in dystonia. In focal task specific-dystonia of the hand, electroencephalography (EEG) and magnetoencephalography (MEG) studies have shown a reduction in movement-related desynchronization in the alpha and beta (10–30 Hz) frequency band in dystonic patients (Toro et al. 2000; Kristeva et al. 2005). One key, unanswered issue is if the abnormal desynchronization is specific to the dystonic posturing muscles or is a generalized mechanism observed when muscles other than the dystonic muscles are used in the task. In addition, it is not established if movement-related abnormal desynchronization is also seen in patients with dystonic tremor (DT). In recent studies, we have used a method of EEG analysis (Bigdely-Shamlo et al. 2013) to study the cortical dynamics associated with voluntary movement in normal subjects and patients with essential tremor and Parkinson’s disease (Ofori et al. 2015; Chung et al. 2017; Chung et al. 2018; Roy et al. 2019). This technique allows high-density EEG (HD-EEG) data to be analyzed as a 4D cortical imaging modality with near-cm scale spatial resolution (Ofori et al. 2015).

Here we describe the cortical dynamics of voluntary movements of 2 cohorts of patients with dystonia (cervical dystonia [CD] cohort; DT cohort) and control individuals during the performance of different visually guided motor control tasks. Our goals are to: 1) better understand if the abnormal cortical dynamics are specific to the dystonic posturing muscles or represent a generalized cortical dysfunction and 2) determine if the abnormal cortical dynamics occur in patients that have DT. The initial cohort of subjects participated in Study 1 and Study 2, and in Study 3 a different cohort of subjects participated. In the first cohort, cortical dynamics were examined in association with head turning movements (Study 1) and upper limb planar movements (Study 2) in patients with isolated focal CD and normal controls. Whereas the understanding of upper limb planar movements is robust (Georgopoulos et al. 1992; Shadmehr et al. 1993; Caminiti et al. 1996; Moran and Schwartz 1999; Ofori et al. 2015), established literature of head movements in normal humans has been limited (Prudente et al. 2016). For instance, the neck muscle representation within the primary motor cortex (and whether neck muscles are controlled ipsilaterally or bilaterally) is still an area of debate (Prudente et al. 2015). In patients with CD, the neck musculature is directly involved in the dystonic head and neck postures. Thus, Study 1 and Study 2 explore if the cortical pathophysiology associated with head movements and upper limb movements represents deficits specific to the musculature involved in the task, or if the cortical pathophysiology of CD only occurs during head movements.

In Study 3, a cohort of DT was compared with a control group. While it is recognized that rhythmic tremulous movements can co-occur in patients with dystonia (Merola et al. 2019; Chen et al. 2020), the underlying pathophysiology remains poorly understood (Kirke et al. 2017). In Study 3, a different task was used, a visually guided grip force task (DeSimone et al. 2019), to study the movement-related cortical dynamics of patients whose dystonia was most severe in a body region other than the tested right upper extremity. The grip force task was selected, because it has been shown to provide a robust measure of cortical dynamics (Roy et al. 2019) and represents a steady state task rather than the ballistic tasks in Study 1 and Study 2. The grip force task was considered a task that would allow more focus on a group of patients with DT.

Across Studies 1, 2, and 3, we tested 2 overall hypotheses. The first hypothesis tested is that abnormal cortical desynchronization will occur in CD during the performance of different tasks, including head turning movements and upper limb movements. Because increased motor cortex excitability is a feature of dystonia, we expect reduced desynchronization. The second hypothesis tested is that patients with and without DT will display abnormal cortical desynchronization. If these hypotheses are confirmed, these observations would provide new evidence that abnormal cortical desynchronization is a general aspect of the pathophysiology of patients with dystonia and occurs also in patients with DT.

## Methods

Two different cohorts of dystonia patients and controls were recruited to participate in 3 experimental tasks. The first cohort was studied with 2 separate experiments (referred to as Study 1 and Study 2) and the second cohort was studied with a third experimental task (Study 3) as shown in Table 1.

**Table 1:** Demographics of Study 1, 2, and 3 cohorts

Measure	Study 1 and 2 group		Study 3 group	
	CD	HC	DT	HC
Sample size	15	17	23	20
Sex	6 M—9 F	7 M—10 F	6 M—17 F	11 M—9 F
Age (years)	65.4 (10.0)	66.8 (9.4)	63.7 (8.3)	62.9 (8.4)
MoCA	26.1 (3.2)	27.1 (2.8)	25.5 (3.0)	27.9 (1.5)
BFMDRS	11.1 (4.0)	-	13.4 (6.4)	-
Disease duration (years)	7.3 (8.0)	-	6.4 (6.7)	-

Note: Mean (SD).

### Subjects

For Study 1 and Study 2, 15 patients with CD and 17 healthy controls (HC) were recruited and matched for age. The 2 groups had a similar sex ratio. For Study 3, 23 patients with DT and 20 HCs were recruited and matched for age (Table 1). In Study 3, the 2 groups did not have the same sex ratio and a Pearson’s Chi-squared test showed a trend toward significance. We explored the effect of sex on EEG activity in this study and found there to be a significant effect below 7 Hz for a brief period after movement onset (Supplementary Fig. 1); however, the primary findings of this study were above this range.

For all 3 studies, CD or DT was diagnosed by movement disorder specialists based on established criteria, dystonia was assessed using the Burke–Fahn–Marsden Dystonia Rating Scale (BFMDRS) and cognition was quantified using the Montreal Cognitive Assessment (MoCA). Prior to all experimental testing, participants provided informed consent. All procedures were approved by the local institutional review board.

### Experimental Design and Task

**Figure 1:**
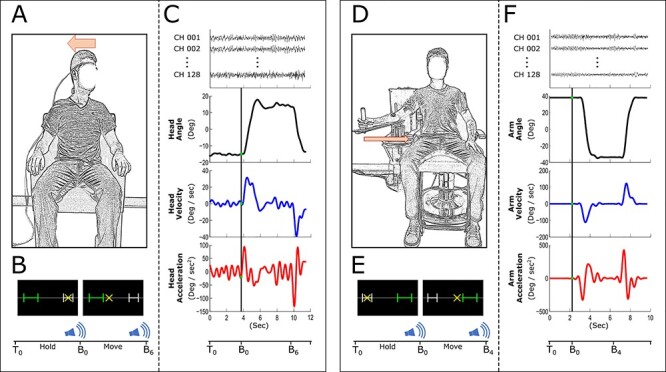
Experimental setup 1 (*A*–*C*). An illustration of a participant, with sensors, holding their head at the starting position prior to turning their head to the other side (*A*). We illustrate the visual feedback displayed on a monitor to the participant (*B*). The gray bracket on the display indicates the starting position, the green bracket indicates the target position, and the yellow “X” indicates the current rotation of the participant’s head. T0, B0, and B6 are the time at beginning of the trial, when the first auditory signal to move is presented and when the second auditory signal to move back to the starting position is presented, respectively. We show an example head rotation trace (black), along with derivatives (blue and red), and EEG channel data (top) (*C*). Experimental setup 2 is shown in *D*–*F*. An illustration of a participant with their right arm resting on a cantilevered beam while holding the manipulandum (*D*). We illustrate the visual feedback displayed on a monitor to the participant (*E*). The gray bracket on the display indicates the starting position, the green bracket indicates the target position, and the yellow “X” indicates the current position of the participant’s arm. T0, B0, and B4 are the time at beginning of the trial, when the first auditory signal to move is presented, and when the second auditory signal to move back to the starting position is presented, respectively. We show an example arm movement trace (black), along with derivatives (blue and red), and EEG channel data (top) (*F*). These paradigms were performed by the CD group and healthy controls.

In Study 1, patients with CD and controls performed a head turning task that directly engaged the muscles involved in producing symptomatic dystonia in the CD group. Subjects sat upright in a chair while two 3D magnetic sensors (Ascension 3D Guidance trakSTAR, TRACKLAB) were attached to the forehead, midway between the supraorbital ridge and hairline, and the chest, an inch below the jugular notch. After digitizing body segments (back of head, C7, and T12), the motion capture system was inspected to ensure correct tracking of subjects’ full range of head motion. There were 2 different head movement conditions: 1) “right to left,” where the subject’s head started with 15° of rotation to the right of center and moved to −15° of rotation to the left of center and 2) “left to right,” where the subject’s head started with −15° of rotation to the left of center and moved to 15° of rotation to the right of center. Visual feedback of the subject’s angular position in the horizontal plane was provided through a 30″ computer monitor (Dell UltraSharp U3011, Dell Co.) that displayed a starting position, a target position, and a cross-shaped cursor that showed the head rotation position (Fig. 1*A*,B). The starting position (15° to the right or left of center, depending on condition) was represented by a white, 1-cm stationary bracket and the target position was represented by a green, 3.5-cm bracket. The starting and target bracket were 15 cm apart (Fig. 1*A*,*B*). The time allotted for each trial was 15 s. After a 7-s baseline in which the subject’s head was at the starting position, a 400-Hz auditory beep cued subjects to begin the movement. During the subsequent 6-s movement period, subjects were instructed to move their head to the target position as fast and accurately as possible from the starting position and maintain the cross-shaped cursor in the target position. A second auditory beep cued the final 2-s period in which subjects were instructed to return the cursor to the starting position at a comfortable pace.

Subjects performed 50 trials in each condition for 100 total trials. The 2 head movement conditions were alternated and pseudorandomized across subjects. EEG data were simultaneously collected with the rotation data and time synchronized (Fig. 1*C*).

In Study 2, subjects performed a ballistic arm movement task that did not involve muscles clinically affected by dystonia in the CD group (e.g., patients had CD, but no limb dystonia). The experiment setup was similar to prior work (Ofori et al. 2015). Subjects sat upright in a chair looking at a monitor directly ahead that would display visual feedback during their task performance while their right arm was supported by a cantilevered beam attached to a custom-made manipulandum. The beam was attached to an angle transducer (Trans-Teck) that allowed for 110° of rotation in the horizontal plane. There were 2 different arm movement conditions: 1) “right to left,” where the manipulandum started with 36° of rotation to the right of center and moved to −36° of rotation to the left of center and 2) “left to right,” where the manipulandum started with −36° of rotation to the left of center and moved to 36° of rotation to the right of center. Subjects were instructed to perform rapid and accurate arm movements. Visual feedback of the subject’s angular position in the horizontal plane was provided through a 30″ computer monitor that displayed a starting position, a target position, and a cross-shaped cursor that showed the arm rotation position (Fig. 1*D*,*E*). The starting position (36° to the right or left of center, depending on condition) was represented by a white, 1-cm stationary bracket and the target position was represented by a green, 3.5-cm bracket. The green bracket represented a 6° range, giving subjects *a* ± 4% of full-range tolerance for the task. The starting and target bracket were 15-cm apart (Fig. 1*D*,*E*). The time allotted for each trial was 12 s. After a 5-s baseline in which the manipulandum was at the starting position, a 400-Hz auditory beep cued subjects to begin the movement. During the subsequent 4-s movement period, subjects were asked to move the manipulandum to the target position as fast and accurately as possible from the starting position and maintain its position there. A second auditory beep cued the final 3-s period in which subjects were instructed to return the cursor (and manipulandum) to the starting position at a comfortable pace. Subjects were asked to perform 50 trials in each condition, for 100 total trials. The 2 arm movement conditions were alternated and pseudorandomized across subjects. EEG data were simultaneously collected with the rotation data and time synchronized (Fig. 1*F*).

**Figure 2:**
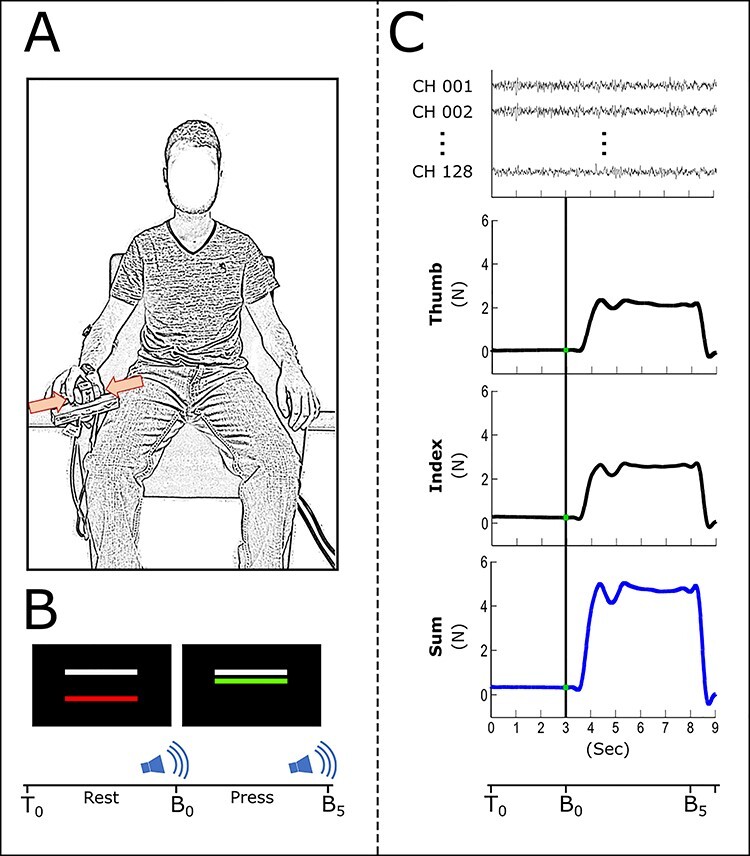
Experimental setup 3 is shown. An illustration of a participant holding the load cells between the thumb and index finger (*A*). We illustrate the visual feedback displayed on a monitor to the participant (*B*). The white bar is the target force level and is fixed in place for a given trial. The color bar is the participant’s current force level. The bar is red during a rest period and turns green to signal force production. T0, B0, and B5 are the time at beginning of the trial, when the first auditory signal to press is presented and when the second auditory signal to release is presented, respectively. We show example force traces from both fingers (black), the sum of these forces (blue), and EEG channel data (top) (*C*). This paradigm was performed by the DT group and healthy controls.

In Study 3, subjects performed a pinch grip task using the right hand that did not involve muscles clinically affected by dystonia in the DT group (e.g., most patients had CD but no dystonia in the hand). The experimental setup was similar to prior work (Roy et al. 2019). Subjects sat upright in a chair looking at a monitor directly ahead that would display feedback on their task performance (Fig. 2*A*,*B*). They were asked to produce force on a pair of load cells attached to the right-side armrest of the chair using only the index finger and thumb of the right hand. The monitor displayed a force bar and a target force bar. Subjects pressed on the load cells to make the force bar rise and would hold a force level matching the target force bar, which was set to 15% of their maximum voluntary contraction. There were 2 different visual feedback conditions: high gain and low gain. Feedback was controlled by manipulating the visual angle. These angles were 0.039° for low gain and 6.9° for high gain, consistent with prior work (Coombes et al. 2010). During high gain, the force bar appeared higher on the screen and would be perceived as being more sensitive to minute force changes, ultimately providing a greater level of information on performance. The experiment consisted of 5 blocks of 10 trials for each level of feedback, for a total of 100 trials. Each trial lasted 15 s with a 10-s resting period and a 5-s pinch period. Low and high visual feedback blocks were alternated and pseudorandomized across subjects. During the rest period, the force bar appeared red and was locked in place at a zero-force position. After 10 s, the force bar would change to green, allowing the subject to produce force for 5 s. Then another rest period would begin. The target force bar was held constant during the rest period and trial (Fig. 2*B*). During each trial, force and EEG data were recorded and time synchronized (Fig. 2*C*).

### Data Acquisition

The MotionMonitor (Innovative Sports Training, Inc.) system was used to synchronize data in real time from EEG and kinematic (or kinetic) recording systems.

#### Behavioral Data

Head motion data in Study 1 were collected with 3D magnetic sensors. Arm motion data in Study 2 were collected with an angle transducer attached to a manipulandum. Force data in Study 3 were collected with 2 opposing load cells attached to a grip apparatus that facilitated a pinch grip task. Each load cell was amplified using a Coulbourn amplifier and sampled at 2000 Hz using a 16-bit A/D converter (Measurement Computing).

#### E‌EG Data

EEG data were collected with the ActiveTwo system (Biosemi) using 128 Ag–AgCl electrodes. These electrodes were connected to a cap with a preconfigured montage that covered the entire scalp. The signal was amplified through the electrode at the source with an output impedance of <1 Ω and sampled at 2048 Hz.

Electrical potentials were recorded between each electrode and the Common Mode Sense (CMS) active electrode and the Driven Right Leg (DRL) passive electrode. The CMS and DRL electrodes were located at the center of the cap in relation to the recording electrodes and were used to drive the average potential of the subject as close as possible to the AD-box reference potential. The electrode offsets, a moving average of the voltage measured between the CMS and each active electrode, were checked during the experiment to be within the acceptable range of below 40 μV. These offsets served as an indirect measure of impedance tolerance.

### Data Processing

#### Behavioral Data Processing

For Study 1 and Study 2, kinematic data were low-pass filtered at 2 Hz and the following 5 measures were computed in time: peak displacement, peak velocity, peak acceleration, peak deceleration, and standard deviation (SD) of acceleration. A span of movement was defined as the moment surpassing 5% maximum velocity to the moment returning back to 5% maximum velocity. All measures were computed within this span.

For Study 3, kinetic data were low-pass filtered at 2 Hz and the following 4 measures were computed during the steady-state phase of the force task: average mean force, between trial SD of mean force, average within-trial-SD-of-force, and between trial SD of within-trial-SD-of-force. These measures were computed over a window defined as the middle 60% of time between the rising and falling edges of force production.

#### E‌EG Data Processing

EEG data were processed using custom EEGLAB procedures developed in previous work (Roy et al. 2019). For Study 1 and Study 2, an epoch of data was defined as the time period 1.5 s before movement onset to 3 s after movement onset. For Study 3, an epoch of data was defined as the time period 1.5 s before force production to 4 s after force production. EEG data were band-pass filtered between 1 and 70 Hz, and channels with artifact were rejected and interpolated using the adjacent channels. Any trial with more than 10 rejected channels or showing large absolute values as defined by EEGLAB’s joint-probability artifact detector (Delorme and Makeig 2004) was deleted. All channels were re-referenced to the global average across channels, and 60-Hz line noise was removed (see Notes). The data were then downsampled to 250 Hz.

For each participant, epochs were concatenated across conditions. Independent components (ICs) and IC weights were calculated using EEGLAB’s runica procedure. For each IC, a corresponding dipole was computed using EEGLAB’s DIPFIT function. Dipoles were excluded if they were located outside the MNI brain, or if their activity did not resemble a dipolar distribution (residual variance >20%) (Chung et al. 2018). Measure projection analysis (MPA) was conducted to sort subject-specific dipoles across groups and conditions into a number of spatially distinct domains (Bigdely-Shamlo et al. 2013) based on their spatial proximity and common information. The information in a dipole is underlying brain activity measured as an event-related spectral perturbation (ERSP). An ERSP represents a time-frequency plot that is normalized by the mean baseline spectrum (Makeig 1993). The baseline was the time period spanning from the beginning of the epoch (−1.5 s) up to movement onset (0 s).

### Statistical Analysis

#### Behavioral Statistical Analysis

The following procedure was done separately for all studies using the output from the behavioral data processing. For each measure, a null hypothesis of equal variance was tested using Levene’s test at each level of the condition. If the between-group variances were found to be significantly different in either condition level, a nonparametric test was used instead of an analysis of variance. Specifically, the Mann–Whitney *U* test was used for comparing group differences and the Wilcoxon signed-rank test was used for comparing condition differences. Significance was set an α = 0.05, and *P*-values were corrected for the false discovery rate (Benjamini and Hochberg 1995).

#### E‌EG Statistical Analysis

The following procedure was done separately for all studies using the output from the EEG data processing step. For each domain, a mean ERSP was computed per subject per condition using dipoles that contributed to the domain. Then, a mean group ERSP and a mean condition ERSP were computed for each domain by averaging together the corresponding subjects and conditions respectively. The dimensions of each mean ERSP matrix were 200 cells evenly divided along the epoch time axis and 100 cells divided along the frequency axis representing 1–60 Hz in log space.

The entire frequency range of the ERSP (1–60 Hz) was evaluated for significant differences within the time window from movement onset (0 ms) to the end of the ERSP time span. At each cell within the previously described time-frequency range, a *t*-test was used to detect group effects, and a matched-pairs *t*-test was used to detect condition effects. In both cases α was set to a level of 0.05. To address the multiple comparisons problem, cluster-based permutation tests (Maris and Oostenveld 2007) were performed over the entire ERSP at α = 0.05.

## Results

### Behavior Analysis

For Study 1, there were no significant differences between CD and controls for any of the 5 kinematic measures (Table 2). There was a significant difference in SD of acceleration between the directions of head movement (*P* = 0.003; Table 2). Subjects demonstrated less variation in acceleration when turning their head toward the right.

**Table 2:** Kinematic and kinetic parameters across studies

Study 1: head turning measures	CD		HC		Group	Direction
	Left	Right	Left	Right	*P* value	*P* value
Peak displacement (deg)	30.7 (1.8)	29.2 (4.0)	30.5 (0.7)	30.8 (0.8)	0.817	0.527
Peak velocity (deg/s)	51.4 (17.4)	49.9 (14.3)	51.5 (9.9)	53.2 (11.3)	0.817	0.678
Peak acceleration (deg/s^2^)	204.8 (86.6)	206.8 (78.3)	193.6 (53.4)	206.7 (59.7)	0.817	0.411
Peak deceleration (deg/s^2^)	−158.3 (80.8)	−167.1 (81.4)	−137.5 (40.0)	−148.7 (56.1)	0.817	0.411
SD of acceleration (deg/s^2^)	33.3 (21.0)	31.0 (24.9)	24.4 (14.0)	21.4 (11.6)	0.590	0.003
**Study 2: arm movement measures**	**CD**		**HC**		**Group**	**Direction**
	**Left**	**Right**	**Left**	**Right**	***P* value**	***P* value**
Peak displacement (deg)	72.1 (1.4)	71.9 (0.8)	71.6 (0.7)	71.8 (0.5)	1	0.324
Peak velocity (deg/s)	83.7 (23.4)	85.6 (23.1)	82.1 (25.8)	78.3 (21.1)	1	0.718
Peak acceleration (deg/s^2^)	228.9 (101.1)	218.6 (92.0)	219.6 (105.1)	200.1 (77.1)	1	0.237
Peak deceleration (deg/s^2^)	−158.9 (84.7)	−161.4 (77.1)	−153.6 (91.3)	−129.0 (60.8)	1	0.260
SD of acceleration (deg/s^2^)	3.5 (2.2)	3.1 (1.5)	3.0 (2.0)	2.9 (1.2)	1	0.498
**Study 3: pinch grip measures**	**DT**		**HC**		**Group**	**Gain**
	**High gain**	**Low gain**	**High gain**	**Low gain**	***P* value**	***P* value**
Average mean force (*N*)	15.0 (0.0)	19.8 (11.4)	15.0 (0.0)	18.2 (3.5)	0.655	<0.001
Between trial SD of mean force	2.4 (0.9)	4.3 (4.2)	1.4 (0.8)	2.8 (1.8)	0.042	<0.001
Average within-trial-SD-of-force	0.9 (0.7)	1.4 (1.4)	0.4 (0.2)	0.8 (0.4)	0.042	<0.001
Between trial SD of within-trial-SD-of-force	0.7 (0.6)	1.1 (1.0)	0.4 (0.6)	0.7 (0.6)	0.217	0.023

Note: Mean (SD).

For Study 2, there were no significant differences between CD and controls for any of the 5 kinematic measures studied (Table 2). There were also no differences between the conditions, meaning no preferential movement when moving their arm toward the right or left.

For Study 3, 2 of the 4 force measures calculated were significantly different between DT and controls (Table 2). DT subjects showed higher between trial SD of mean force (*P* = 0.042) and higher average within-trial-SD-of-force (*P* = 0.042). This means that when trying to reach the same force level between trials, the DT group was less precise, and when holding a constant level of force, the DT group showed more difficulty maintaining that force. All 4 measures were significantly different when comparing high and low visual gain. During low visual gain subjects produced more average mean force, more between trial SD of mean force, more average within-trial-SD-of-mean-force, and more between trial SD of within-trial-SD-of-force. In general, this means that during low visual gain subjects tended to produce more force than necessary while being less consistent with how much force they produced within and between trials.

### E‌EG Analysis

#### Study 1

MPA yielded 2 domains (Supplementary Fig. 2*A*): a parietal area (sensory domain) and a motor area (motor domain).

##### Motor domain

**Figure 3:**
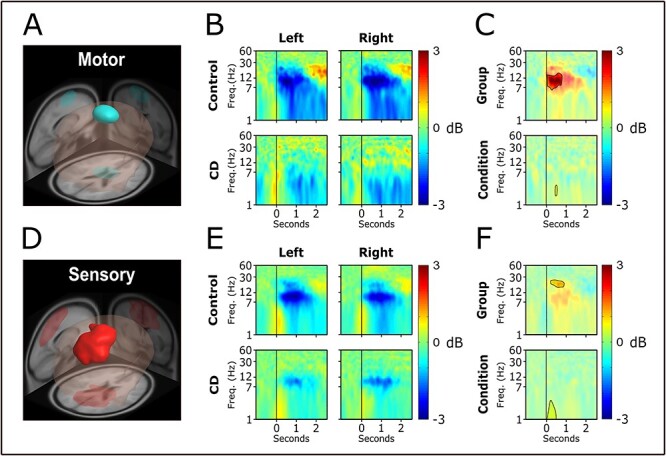
EEG analysis results 1. We show the location and size of the motor and sensory domains as revealed by MPA (*A* and *D*). ERSP time–frequency plots shown by condition (left and right) and group (control and CD) for each of the domains (*B* and *E*). A line at time equals zero is placed to show when the head rotation began. Difference plots for group (CD−control) and condition (left−right) are shown along with the results of the statistical analysis (*C* and *F*). Significant areas are bright. Nonsignificant areas are semi-transparent.

The domain (Fig. 3*A*) overlapped the premotor and supplementary motor regions of the brain (Table 3). Figure 3*B* shows the average ERSP by condition and group. In the control group, there was a visible decrease of power in the alpha (8–12 Hz) and beta bands (13–30 Hz) for the first 2 s of movement and then an increase of power in the beta band afterward, relative to baseline (Fig. 3*B*). This activity was not as clearly visible in the CD group. Figure 3*C* shows that the difference was statistically significant in the alpha and low beta range within the first second of movement. A difference was found between conditions in the lower frequencies at around 0.5 s. A burst in activity can be seen in the theta (4–8 Hz) range during movement onset, but no differences were found between groups or conditions.

**Table 3:** Domain anatomical locations across studies

Study 1								
Sensory			Motor					
Area	Probability	Description	Area	Probability	Description			
BA 31	0.41	Posterior cingulate	BA 6	1.00	Premotor and supplementary			
BA 7	0.30	Somatosensory						
BA 5	0.10	Somatosensory						
BA 4	0.07	Primary motor						
Study 2								
Sensorimotor			R-Motor			L-Motor		
Area	Probability	Description	Area	Probability	Description	Area	Probability	Description
BA 7	0.34	Somatosensory association	BA 6	0.58	Premotor and supplementary	BA 6	0.64	Premotor and supplementary
BA 5	0.15	Somatosensory association	BA 4	0.19	Primary motor	BA 4	0.19	Primary motor
BA 31	0.14	Posterior cingulate	BA 3	0.14	Primary somatosensory	BA 3	0.11	Primary somatosensory
BA 4	0.13	Primary motor						
Study 3								
R-Sensorimotor			M-Sensorimotor			L-Sensorimotor		
Area	Probability	Description	Area	Probability	Description	Area	Probability	Description
BA 6	0.58	Premotor and supplementary	BA 4	0.28	Primary motor	BA 6	0.44	Premotor and supplementary
BA 3	0.15	Primary somatosensory	BA 6	0.27	Premotor and supplementary	BA 4	0.23	Primary motor
BA 4	0.15	Primary motor	BA 5	0.18	Somatosensory	BA 3	0.20	Primary somatosensory
			BA 3	0.17	Primary somatosensory			
Sensory								
Area	Probability	Description						
BA 7	0.50	Somatosensory						
BA 39	0.26	Angular gyrus						
BA 31	0.09	Posterior cingulate						

##### Sensory domain

The domain (Fig. 3*D*) overlapped the posterior cingulate and somatosensory association regions of the brain (Table 3). Figure 3*E* shows the average ERSP by condition and group. For both groups and conditions there was reduced power in the alpha and beta bands during movement, relative to baseline (Fig. 3*E*). Although there appears to be lower alpha-band power in the control group, it was not statistically different (Fig. 3*F*). The brief period of beta band desynchronization seen in the control group, but not in the CD group, was statistically different. A significant difference in the lower frequencies was found between conditions, related to the burst in activity that can be seen in the theta range during movement onset.

### Study 2

MPA yielded 3 domains (Supplementary Fig. 2*B*): a sensorimotor area (sensorimotor domain), a left motor area (L-motor domain), and a right motor area (R-motor domain).

####  

##### R-Motor domain

**Figure 4:**
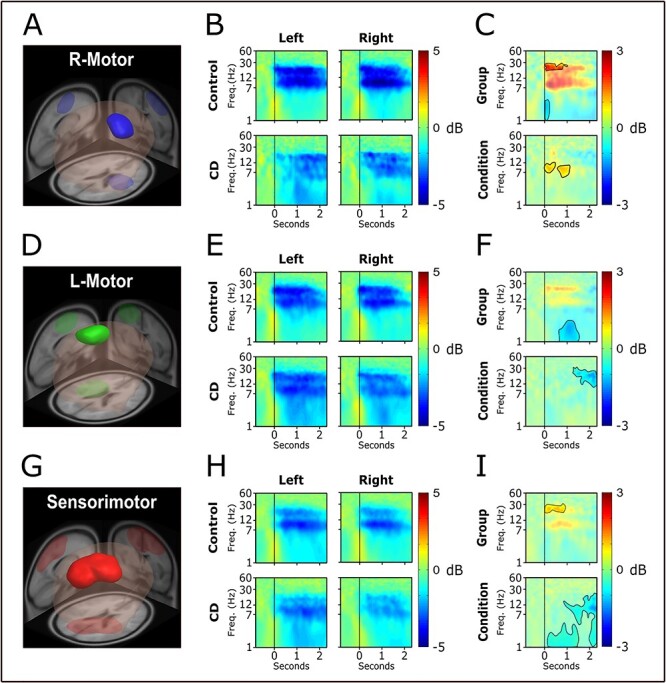
EEG analysis results 2. We show the location and size of the R-motor, L-motor, and sensorimotor domains as revealed by MPA (*A*, *D*, and *G*). ERSP time–frequency plots shown by condition (left and right) and group (control and CD) for each of the domains (*B*, *E*, and *H*). A line at time equals zero is placed to show when the arm movement began. Difference plots for group (CD−control) and condition (left−right) are shown along with the results of the statistical analysis (*C*, *F*, and *I*). Significant areas are bright. Nonsignificant areas are semitransparent.

The domain (Fig. 4*A*) overlapped the primary motor, premotor, and supplementary motor regions (Table 3) in the right hemisphere of the brain, which was ipsilateral to the task arm. Figure 4*B* shows the average ERSP by condition and group. For both groups and conditions, there was reduced power in the alpha and beta bands during movement, relative to baseline (Fig. 4*B*). Alpha- and beta-band power appear lower in the control group, and this difference was statistically different in the high beta band range (Fig. 4*C*). Desynchronization when turning the arm toward the right was somewhat stronger in the alpha band than when turning left. A theta band burst during movement onset was also present, with statistical differences between groups.

##### L-Motor domain

The domain (Fig. 4*D*) overlapped the primary motor, premotor and supplementary motor regions (Table 3) in the left hemisphere of the brain, which was contralateral to the task arm. Figure 4*E* shows the average ERSP by condition and group. For both groups and conditions there was reduced power in the alpha and beta bands during movement, relative to baseline (Fig. 4*E*). Alpha- and beta-band power appears slightly lower in the control group, but it was not statistically significant (Fig. 4*F*). Some differences in the lower frequencies were found between groups, and a difference was found between 1 and 2 s in the beta band between conditions. A theta band burst during movement onset was also present, but no differences were found between groups or conditions.

##### Sensorimotor domain

The domain (Fig. 4*G*) overlapped the somatosensory association, posterior cingulate, and primary motor regions of the brain (Table 3). Figure 4*H* shows the average ERSP by condition and group. For both groups and conditions there was reduced power in the alpha and beta bands during movement, relative to baseline (Fig. 4*H*). The difference between groups was significant in the high beta range (Fig. 4*I*). A theta band burst during movement onset was also present, but no differences were found between groups. There was also a widespread difference between conditions that was found to be statistically significant.

#### Study 3

MPA yielded 4 domains (Supplementary Fig. 2*C*): a right sensorimotor area (R-sensorimotor domain), a medial sensorimotor area (M-sensorimotor domain), a left sensorimotor area (L-sensorimotor domain), and a parietal area (sensory domain).

##### M-Sensorimotor domain

**Figure 5:**
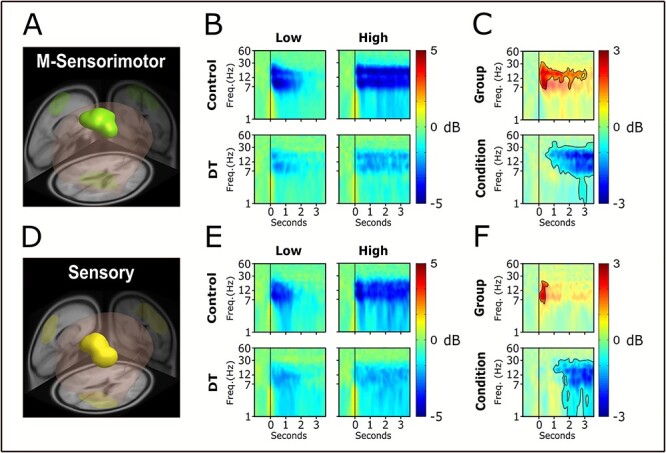
EEG analysis results 3. We show the location and size of the M-sensorimotor and sensory domains as revealed by MPA (*A* and *D*). ERSP time–frequency plots shown by condition (low and high) and group (control and DT) for each of the domains (*B* and *E*). A line at time equals zero is placed to show when grip force production began. Difference plots for group (DT−control) and condition (high−low) are shown along with the results of the statistical analysis (*C* and *F*). Significant areas are bright. Nonsignificant areas are semitransparent.

The domain (Fig. 5*A*) overlapped the primary motor, premotor, supplementary motor, and somatosensory regions of the brain (Table 3). Figure 5*B* shows the average ERSP by condition and group. For both groups and conditions there was a decrease of power in the alpha and beta bands, relative to baseline. Beta- and alpha-band power appears lower in the control group (Fig. 5*B*). This decrease in power persisted throughout the grip force production during the high visual gain condition, but only part way through the grip force production during the low visual gain condition. Figure 5*C* shows that the statistical difference between groups was significant in most of the beta band and for a small duration in the alpha band. The differences between conditions were also statistically significant, especially after the first second of grip force production. The theta burst at force production onset was present, with no differences between groups or condition.

##### Sensory domain

The domain (Fig. 5*D*) overlapped the somatosensory, angular gyrus, and posterior cingulate regions of the brain (Table 3). Figure 5*E* shows the average ERSP by condition and group. Much like in the M-sensorimotor domain, there was a decrease of power in the alpha and beta bands, relative to baseline (Fig. 5*E*). Alpha and beta power appears to be lower for the control group, and the difference was statistically significant within the first second of the force task (Fig. 5*F*). The condition was statistically different, especially after the first second of grip force production. The theta burst at force production onset was present, with no differences between groups or condition.

The R-sensorimotor and L-sensorimotor domains followed a similar pattern to the sensory domain where there were some small clusters of statistical difference between controls and DT, but there was a large difference between high and low visual gains after the first second of grip force production (Supplementary Fig. 3*C*,*F*).

A summary of the experiments and EEG results is provided in Supplementary Table 1.

## Discussion

The current study examined cortical dynamics during voluntary movements of 2 cohorts of patients with dystonia and controls. In Study 1 and Study 2, head movements and upper limb planar movements were studied in patients with CD and control individuals (Fig. 1). In Study 1 while exploring head movements, it was found that in bilateral motor and sensory domains CD had reduced desynchronization in the alpha and beta bands compared with control individuals (Fig. 3). In Study 2 while exploring upper limb planar movements, the ipsilateral motor and bilateral sensorimotor domains had reduced desynchronization in the upper beta band compared with control individuals, whereas the contralateral motor domain was not different between groups in the beta band (Fig. 4). These findings suggest that sensorimotor cortical desynchronization is abnormal during voluntary head and upper limb movements in CD. Further, the findings suggest that abnormal sensorimotor cortical desynchronization is observed when the movement involves the dystonic posturing muscles (head movement) and when the movement uses muscles not primarily affected by the dystonia (upper limb movement). In Study 3, patients with DT were found to also demonstrate impaired cortical desynchronization (M-sensorimotor domain) in the alpha and beta bands throughout the grip force production (Figs 2 and 5). Collectively, these new findings point to a generalized impairment in sensorimotor cortical desynchronization in the alpha and beta bands during voluntary movements involving both dystonic and nondystonic muscles in patients with CD and patients with DT.

A question that can be raised from these findings is, what is the abnormal cortical desynchronization related to? Two studies using different methods offer some insight on the current findings. In a study by Burciu et al. (2017), the authors used motor task-based fMRI to understand how neuronal activity measured by the blood oxygen level-dependent (BOLD) signal is altered in CD. They observed that in CD the somatosensory cortex BOLD signal decreased, whereas the dorsal premotor and cerebellum lobule VI BOLD signal increased compared with controls. Further, the somatosensory and cerebellar BOLD signal significantly related to the clinical severity of dystonia measured by the BFMDRS. In another study of CD, Corp et al. (2019) used a lesion mapping approach in rare CD patients that present with a lesion. While the lesion location varied across patients, the lesions were generally part of a similar network. In fact, when using a clever approach to identify the connectome network during resting state, the authors identified that all of the lesioned regions had a positive connectivity with the cerebellum, and negative connectivity with the somatosensory cortex. These 2 studies point to the cerebellum and the somatosensory cortex as fundamentally important in CD patients with and without a lesion. Here, Studies 1, 2, and 3 used unique methodology and identified a domain that always included the sensory cortex across all 3 studies. Further, the impaired desynchronization was identified in domains including sensorimotor cortex in the upper beta band in Study 1 and 2, and in the alpha and beta bands in Study 3. These findings suggest that a common pathophysiology in CD is abnormal sensorimotor cortical function that manifests using different neuroimaging and analysis approaches.

Prior studies using electrophysiology and imaging methods point to altered inhibitory circuits in the sensorimotor cortex of patients with dystonia. It is thus possible that the lack of desynchronization relates to altered inhibitory circuits. In a study using EEG where participants performed a task in the affected hand for focal dystonia, the authors found that the event-related desynchronization within a 20–30 Hz frequency band was reduced in the focal dystonia patients (Toro et al. 2000). A mutual information approach was used as a quantitative measure of linear and nonlinear coupling across EEG channels, and it was determined that focal dystonia patients had reduced mutual information in the beta band (Jin et al. 2011). Several studies using transcranial magnetic stimulation support the work in EEG. When examining motor cortex excitability, intracortical inhibition was studied during wrist movements of patients with upper limb dystonia (Gilio et al. 2003). The authors found that dystonia patients had intracortical inhibition decreases that were impaired, thus a lack of inhibition. Further, transcranial magnetic stimulation studies have identified excessive sensorimotor plasticity and increased motor cortex excitability as key pathological features (Wagle Shukla and Vaillancourt 2014). In a study examining subthalamic deep brain stimulation in CD, the short and long latency afferent inhibition were both improved in patients with dystonia, whereas motor cortex excitability was not affected (Shukla et al. 2018). When comparing the effects of deep brain stimulation on resting-state alpha-band power, it was found that stimulation led to reduced alpha-band power, suggesting that at rest the sensorimotor network is synchronized in patients with dystonia (Miocinovic et al. 2018). The issue of altered inhibitory circuits has been hypothesized to relate to excessive tendency to form associations between sensory inputs and motor outputs (Quartarone et al. 2006). This idea is also supported by imaging studies showing lower than normal gamma-aminobutyric acid levels in the sensorimotor area for patients with focal hand dystonia, suggesting altered inhibitory neurotransmitter levels (Levy and Hallett 2002). Furthermore, a positron emission tomography/computed tomography study (Berman et al. 2018) with CD patients showed increased gamma-aminobutyric acid type A receptor availability within the right precentral gyrus, which is thought to be a compensatory response to loss of inhibition. Future studies may seek to use magnetic resonance spectroscopy to determine neurotransmitter levels along the motor network in this patient population.

Another possible explanation for the consistent patterns of abnormal desynchronization occurring during movements with and without dystonic posturing muscles is that all of these tasks involve timing perception mechanisms. The tasks required participants to switch from acceleration to deceleration and to switch from increasing force output to decreasing it. The switch between behaviors would require a temporal perception of proprioceptive and visual senses. A series of studies have explored temporal discrimination thresholds. A version of this behavioral task requires that a participant determine if consecutive sensory stimuli are either 1 stimulus or 2 stimuli. It has consistently been shown that the somatosensory temporal discrimination threshold is increased in patients with dystonia (Fiorio et al. 2003; Molloy et al. 2003). This fundamental behavioral deficit is considered to be a deficit in temporal sensory processing. Using a paired-pulse somatosensory evoked potential paradigm, the authors found that the dystonic patients had longer somatosensory temporal discrimination thresholds, reduced suppression of cortical and subcortical paired-pulse somatosensory evoked potentials, less spatial inhibition of simultaneous evoked potentials, and a smaller area of the early component of high-frequency oscillations (Antelmi et al. 2017).

In the current study, all of the motor control tasks required sensory processing. In the head movement task, participants had to match an initial angle and then turn the head to a new angle. This requires that a participant monitor the afferent input from muscle proprioceptors and skin stretch receptors, in addition to the visual target (Fig. 1). Similarly, the upper limb movement task required subjects to monitor muscle proprioceptors, skin stretch receptors, and a visual target. Finally, the grip force task required that participants monitor the pressure against the skin surface from mechanoreceptors in the dermal and epidermal layers and a visual target, throughout the entire grip force contraction (Fig. 2). It is thus possible that the consistent patterns of abnormal sensorimotor cortical desynchronization relate to sensory processing deficits, which are required in each of the tasks.

A novel finding in this study was that patients with DT, similar to dystonia patients without tremor, demonstrate altered desynchronization patterns (Fig. 5). This finding is important as there is a specific need to better understand and develop treatments specific to DT (Fasano et al. 2014). While we did not directly compare CD patients with and without tremor, prior studies in CD have shown unique changes in CD patients with DT in terms of somatosensory function and pathophysiology. CD patients with and without tremor have been studied using the tactile discrimination threshold and a proprioceptive acuity paradigm. It was found that both CD groups had impaired tactile discrimination, whereas the CD group with tremor also had deficits in proprioceptive acuity (Avanzino et al. 2020). When examining the firing patterns of globus pallidus neurons in CD patients with and without tremor, the authors observed that burst neurons were more common in the pure dystonia group compared with the CD group with tremor (Sedov et al. 2020). Further, pause neurons were more common in the CD group with tremor compared with the patients without tremor. A limitation of the current study was that we could not directly compare CD patients with and without DT because the participants were involved in different studies. However, the current findings point to both CD patients with and without tremor experiencing impaired sensorimotor cortical desynchronization.

The current study observed group differences between dystonia and control individuals, in both the alpha and beta bands. There did not seem to be a specific preference for a group difference in the alpha or beta bands in terms of differences between groups in the movement and force tasks. This observation is consistent with prior work in the upper limb task, in which healthy adults showed task effects in the alpha and beta bands near the end of the movements (speed and accuracy constraints) (Ofori et al. 2015). In addition, when examining the spatial gain of visual feedback (Chung et al. 2017), these effects led to greater desynchronization in the high-gain condition compared with a low-gain condition that was broadly observed across alpha and beta bands.

There are several other limitations to the current study. The dystonia patients recruited for this study were mainly recruited from a botulinum clinic; thus, there is a bias against patients that choose not to be treated with this method. While we tested patients near the end of their botulinum regimen, we did not control for other treatments and medications they were taking. A future study could control for medications and determine the effects of these treatments on cortical desynchronization. In our study, we used several steps to minimize head movement artifacts; however, it is still possible that these effects were not completely removed and prevented from influencing the EEG signals. One aspect of the data that gave us confidence in our ability to minimalize the effect head movement is that the anatomical locations of the domains were consistent with the hand and head regions confirmed by others (Prudente et al. 2015). When seated in an upright position, participants hold their heads straight, which requires the activation of postural muscles for stabilization. It is possible that some activation of the neck muscles occurred during the upper limb movement. However, due to the location of the domains, we are confident that any coactivation was minimal and did not strongly affect the results. While the current focus was on movement-related desynchronization, there might have been activity several 100 ms before the movement onset. A future study could look for differences in this premovement-related desynchronization. Finally, the 3 tasks of this study were conducted with augmented visual feedback. We do not know if the same results would occur in the absence of this enhanced feedback.

In summary, this study examined CD, DT, and controls in different motor control tasks that involved dystonic posturing muscles and tasks that did not involve dystonic posturing muscles. Using HD-EEG, we observed a general pattern of abnormal sensorimotor cortical desynchronization in the alpha and beta bands that was present across the tasks studied and in both dystonic and nondystonic muscles, suggesting that abnormal sensorimotor cortical desynchronization is a fundamentally abnormal feature of motor control in patients with dystonia. Further, we found for the first time that DT patients also have impaired cortical desynchronization. Our findings provide new insight into the pathophysiology of dystonia and DT.

## Notes

NITRC 2012: CleanLine: Tool/Resource Info. *Conflict of Interest*: None declared.

## Funding

The National Institutes of Health (R01 NS058487, R01 NS075012, and K23 NS092957).

## Supplementary Material

SupFigure1_tgaa048

SupFigure2_tgaa048

SupFigure3_tgaa048

Dystonia_supp_legends_tgaa048

Dystonia_supp_table_tgaa048
