# Supplementary figures and images for: Cortical Oscillations in Cervical Dystonia and Dystonic Tremor
Source: Cereb Cortex Commun. 2020 Aug 20;1(1):tgaa048. doi: 10.1093/texcom/tgaa048 (PMC7503385; doi:10.1093/texcom/tgaa048)

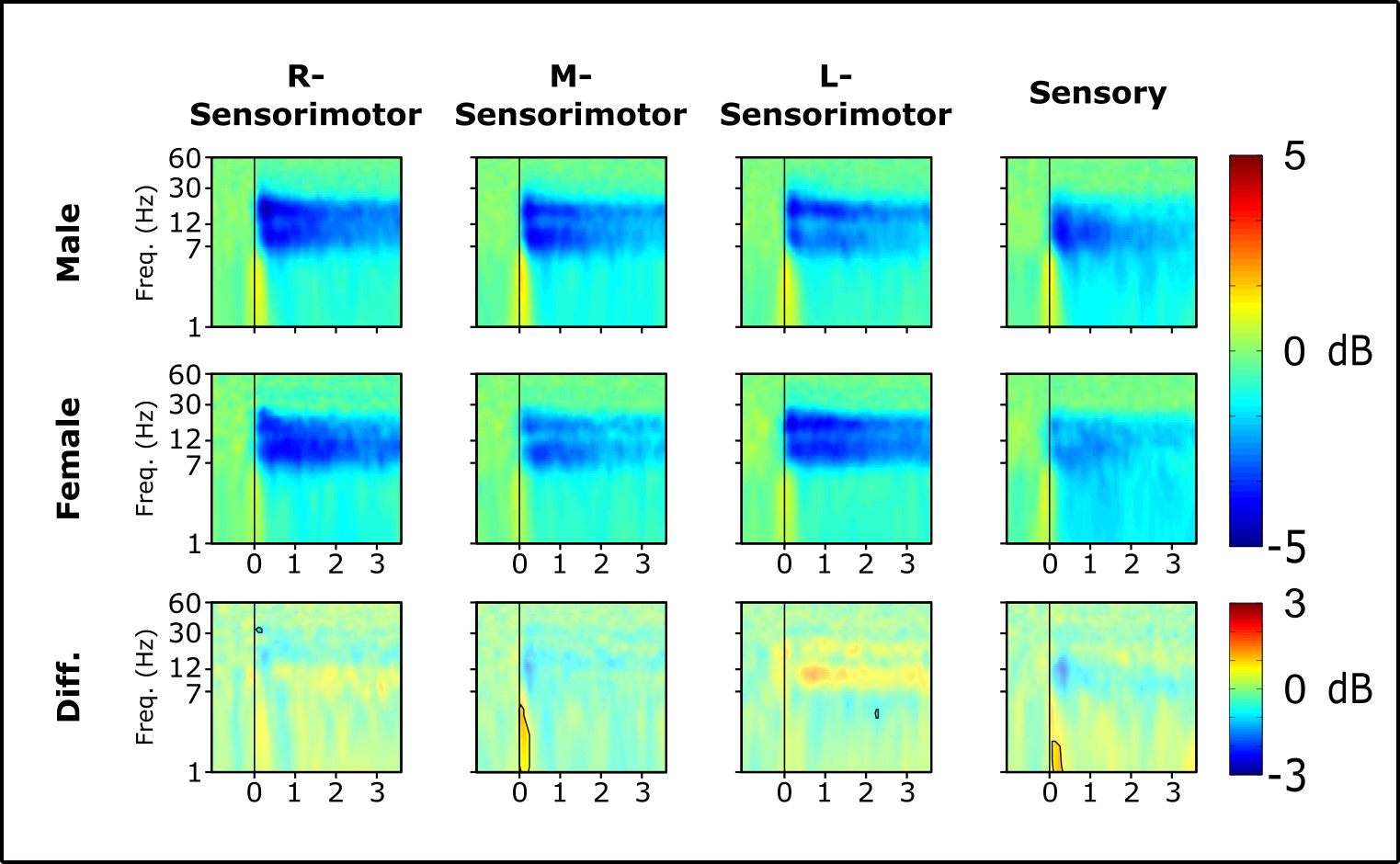

Supplement: SupFigure1_tgaa048 [file supfigure1_tgaa048.jpeg]

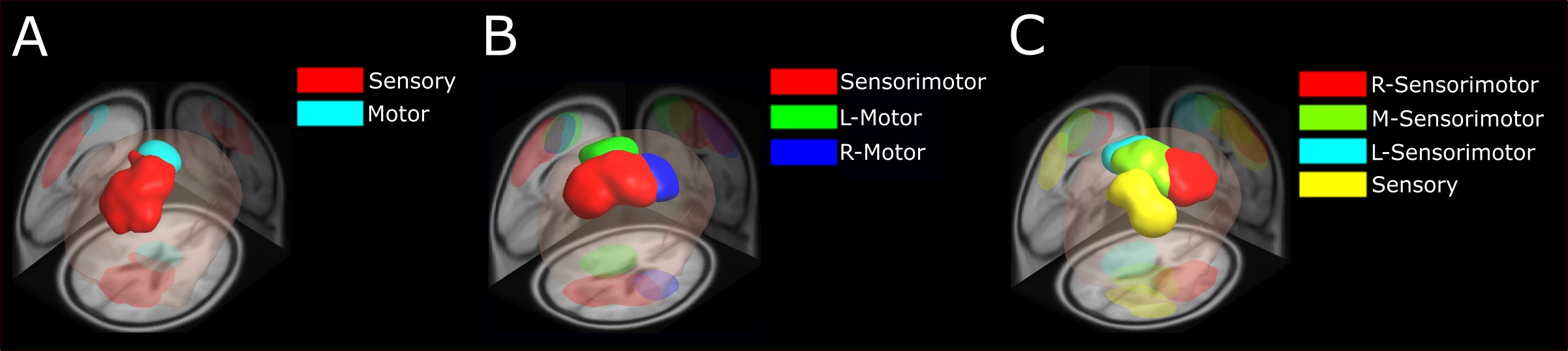

Supplement: SupFigure2_tgaa048 [file supfigure2_tgaa048.jpeg]

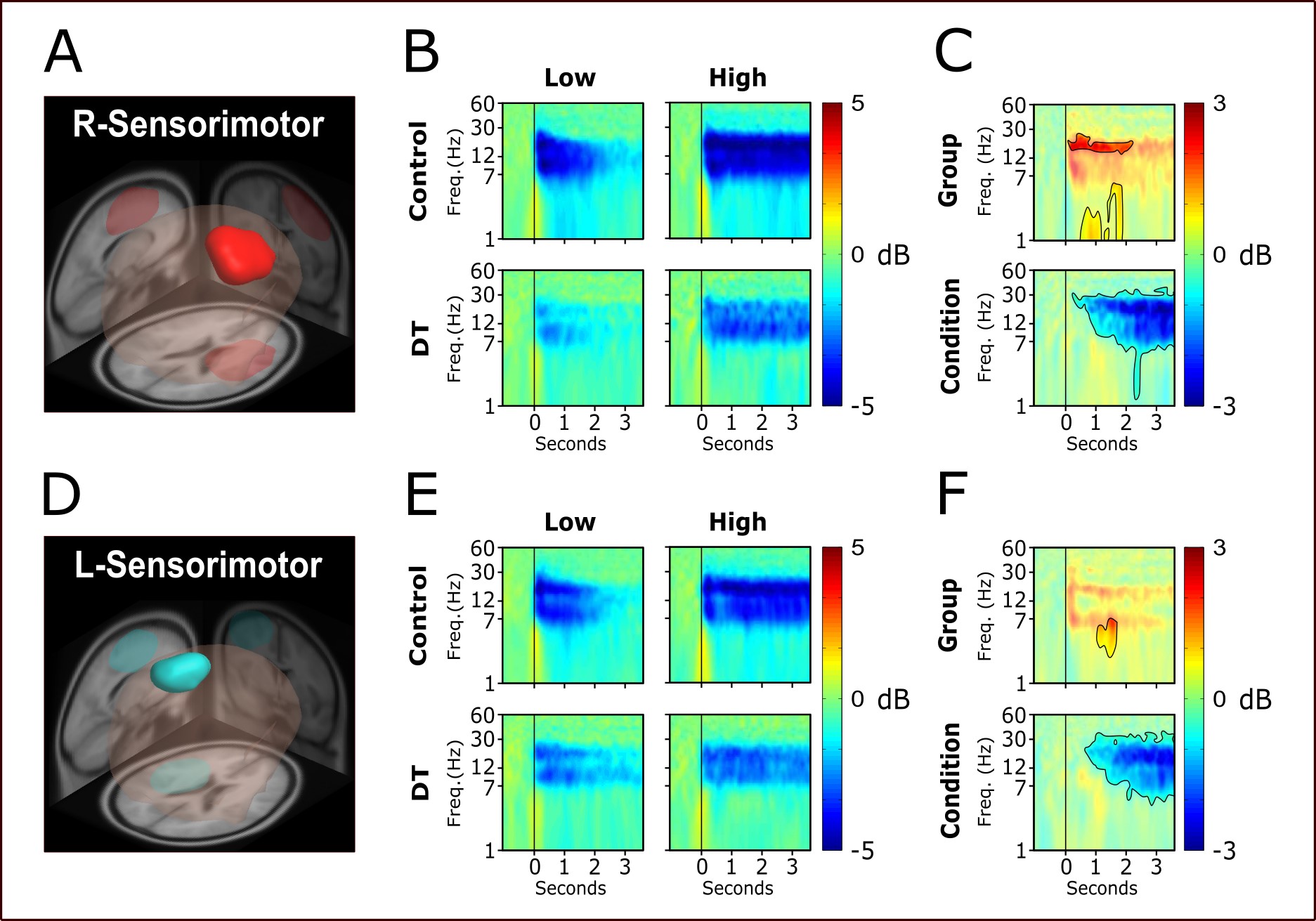

Supplement: SupFigure3_tgaa048 [file supfigure3_tgaa048.jpeg]
