# Supplementary material for: Cortical Oscillations in Cervical Dystonia and Dystonic Tremor
Source: Cereb Cortex Commun. 2020 Aug 20;1(1):tgaa048. doi: 10.1093/texcom/tgaa048 (PMC7503385; doi:10.1093/texcom/tgaa048)
Supplement: Dystonia_supp_legends_tgaa048 [file dystonia_supp_legends_tgaa048.docx]

Supplementary Figure 1: We show the average ERSP plots of dipoles by domain and sex for the dystonic tremor group and healthy controls (Study 3). A line at time equals zero is placed to show when grip force production began. Difference plots for sex (Male - Female) are shown along with the results of the statistical analysis (third row). Regions of statistical significance between sex are enclosed by a black border and the colors are made more vibrant for the reader to identify. For more information on domains and the statistical tests, see Data Processing and Statistical Analysis of the Methods.

Supplementary Figure 2: Domains revealed by MPA. Two domains were revealed for Study 1 (A). They were named Sensory Domain and Motor Domain. Three domains were revealed for Study 2 (B). They were named Sensorimotor Domain, L-Motor Domain and R-Motor Domain. Four domains were revealed for Study 3 (C). They were named Right-Sensorimotor Domain, Medial- Sensorimotor Domain, Left- Sensorimotor Domain, and Sensory Domain.

Supplementary Figure 3: EEG Analysis Results 3. We show the location and size of the R-Sensorimotor and L-Sensorimotor Domains as revealed by MPA (A and D). ERSP time-frequency plots shown by condition (Low and High) and group (Control and DT) for each of the domains (B and E). A line at time equals zero is placed to show when grip force production began. Difference plots for group (DT - Control) and condition (High - Low) are shown along with the results of the statistical analysis (C and F). Significant areas are bright. Non-significant areas are semi-transparent.
