# Supplementary material for: Cortical Oscillations in Cervical Dystonia and Dystonic Tremor
Source: Cereb Cortex Commun. 2020 Aug 20;1(1):tgaa048. doi: 10.1093/texcom/tgaa048 (PMC7503385; doi:10.1093/texcom/tgaa048)
Supplement: Dystonia_supp_table_tgaa048 [file dystonia_supp_table_tgaa048.docx]

|  |  | Study 1 |  | Study 2 |  | Study 3 |
| --- | --- | --- | --- | --- | --- | --- |
|  |  |  |  |  |  |  |
| Subjects |  | Cohort 1: 15 patients with cervical dystonia (CD) and 17 healthy controls (HC) were recruited. Average age 66 years. |  | Cohort 1: 15 patients with cervical dystonia (CD) and 17 healthy controls (HC) were recruited. Average age 66 years. |  | Cohort 2: 23 patients with dystonic tremor (DT) and 20 healthy controls (HC) were recruited. Average age 63 years. |
|  |  |  |  |  |  |  |
| Experimental Task |  | Head Turning: subjects performed 30° head rotations on the horizontal plane. Rotations were made towards the left and right, 50 times each. |  | Arm Movement: subjects performed right arm movements in the horizontal plane by swinging a manipulandum 72° around its axis. Movements were made towards the left and right, 50 times each. |  | Pinch Grip: subjects performed force targeting contractions on a pair of load cells using the index finger and thumb of the right hand. Contractions were performed under augmented visual feedback with a high and low magnification, 50 times each. |
|  |  |  |  |  |  |  |
| EEG Domains |  | Measure Projection Analysis revealed 2 domains: • Motor: BA6 • Sensory: BA31 / BA7 / BA5 / BA4 |  | Measure Projection Analysis revealed 3 domains: • R-Motor: BA6 / BA4 / BA3 • L-Motor: BA6 / BA4 / BA3 • Sensorimotor: BA7 / BA5 / BA31 / BA4 |  | Measure Projection Analysis revealed 4 domains: • R-Sensorimotor: BA6 / BA4 / BA3 • M-Sensorimotor: BA4 / BA6 / BA5 / BA3 • L-Sensorimotor: BA6 / BA4 / BA3 • Sensory: BA7 / BA39 / BA31 |
|  |  |  |  |  |  |  |
| EEG ERSPs |  | • Motor: desynchronization in the alpha (8-12 Hz) and beta (13-30 Hz) bands. Resynchronization in the beta band. Theta (4-8 Hz) burst present at movement onset. CD group lacks the desynchronization and resynchronization. • Sensory: desynchronization primarily in the alpha band, some in the beta band. Theta burst present at movement onset. CD group lacks desynchronization of the beta band. |  | • R-Motor: desynchronization in the alpha and beta bands. Theta burst present at movement onset. CD group presents with weaker beta band desynchronization and theta burst. • L-Motor: desynchronization in the alpha and beta bands. Theta burst present at movement onset. • Sensorimotor: desynchronization in the alpha and beta bands. Theta burst present at movement onset. CD group presents with weaker beta band desynchronization. |  | • M-Sensorimotor: desynchronization in the alpha and beta bands. Theta burst present at force onset. DT group presents with weaker desynchronization over the entire task. • R/L-Sensorimotor: similar pattern to M-Sensorimotor domain with small clusters of statistical significance between DT and HC. • Sensory: desynchronization in the alpha and beta bands. Theta burst present at force onset. DT group presents with weaker desynchronization at the beginning of the task. |
|  |  | BA: Brodmann Area |  |  |  |  |

**Supplementary Table 1. Summary of Studies**
